# Editorial: Emerging applications of text analytics and natural language processing in healthcare

**DOI:** 10.3389/fdgth.2023.1227948

**Published:** 2023-06-23

**Authors:** Khairunnisa Hasikin, Khin Wee Lai, Suresh Chandra Satapathy, Kadir Sabanci, Muhammet Fatih Aslan

**Affiliations:** ^1^ Department of Biomedical Engineering, Faculty of Engineering, Universiti Malaya, Kuala Lumpur, Malaysia; ^2^ Center of Intelligent Systems for Emerging Technology (CISET), Faculty of Engineering, Universiti Malaya, Kuala Lumpur, Malaysia; ^3^ School of Computer Engineering, Kalinga Institute of Industrial Technology, Deemed to Be University, Bhubaneswar, India; ^4^ Department of Electrical and Electronics Engineering, Karamanoglu Mehmetbey University, Karaman, Turkey

**Keywords:** text processing, machine learning (ML), deep learning, Word2Vec analysis, artificial intelligence, LSTM (Long short term memory networks)

**Editorial on the Research Topic** Emerging applications of text analytics and natural language processing in healthcare

Text analytics and natural language processing (NLP) have emerged as powerful tools in healthcare, revolutionizing patient care, clinical research, and public health administration. Over the years, as healthcare databases expand exponentially, healthcare providers, pharmaceutical and biotech industries are utilizing both tools to enhance patient outcomes. In addition, the proliferation of technologies for wearable devices has opened new opportunities to exploit the consumer health data. For example, Khairuddin et al. (1) proposed a multimodal input data combining the text features and numerical data of occupational safety and health management system in reducing workplace injury cases. In addition, the patients’ clinical data were also included with the breast thermal images in the predictive model development of breast cancer detection (2). In this research topic, the publications have been rigorously peer-reviewed by external reviewers with strong background of text analytics and innovations in the NLP research especially in healthcare applications. Publications on this research topic have been subjected to rigorous peer review by external reviewers with a solid background in text analytics and innovations in NLP research, with a focus on healthcare applications.

Among the published works in this Research Topic, three research groups focused on the emerging applications of text analytics and NLP in medical/clinical applications. Zhou et al. (3) proposed an ensemble transfer learning approach to oncological field named Biomedical Named Entity Recognition (NER) in Chinese clinical records. Using an ensemble approach, the researchers combine multiple models to improve the efficacy of the NER system. The first stage of the model consists of the pre-trained Word2Vec and Bidirectional Embeddings Representations to attain domains-specific word embeddings. These are then used as an input to the second stage that consists of Bidirectional Long and Short Time Memory Recurrent Neural Network (BiLSTM) and Linear-chain Conditional Random Field (CRF). By combining multiple models, each with their own strengths and weaknesses, the ensemble seeks to produce more precise and reliable results. In addition, the authors employ augmented domain resources, such as additional labelled data or external oncology-related knowledge sources, to further improve the performance of the NER system.

The other group focus on integrating text analytics on dynamic multimodal feature recognition for mental health assessment (Xu et al.). The research incorporating interactive assessment scales containing voice interaction, video acquisition, and statistical analysis modules with the Depression Anxiety Stress Scales (DASS-21). The proposed method entails capturing and analysing various characteristics from various modalities which include acoustic characteristics of speech, and facial expressions from video recordings. The method attempts to capture a more comprehensive understanding of an individual’s mental state by incorporating multiple modalities. Through dynamic multimodal feature recognition, the identification of patterns, changes, or anomalies that can reveal information about a person’s mental health status were enabled. The study recruited 1,500 participants for this mental health evaluation, in which the facial video model and the audio emotion detection model were separately fed to the convolutional neural network architecture. Then, each output was fed to the LSTM, where multimodal data fuses potentially shared information of each modality and compares emotion recognition results using an enumeration-weighted decision fusion strategy based on statistical rules and probability theory.

Additionally, the other research group solely focused on the natural language processing technology in medical rehabilitation (Wang and Sun). This study highlights the growing uses of text analytics and natural language processing in the healthcare industry, specifically in the context of enhancing physical fitness evaluation and rehabilitation procedures for teenagers who are physically handicapped. According to the findings of the study, it is critical to have policies that are both comprehensive and well implemented in order to meet the requirements of impaired people and to foster their growth. This study investigates the connection between physically challenging exercise and positive mental health in young people who have a physical disability, using NLP technology. The findings provide important information for medical practitioners and policymakers. The findings provide light on the obstacles that are faced by adolescents who are physically challenged and emphasise the need of individualised rehabilitation programmes to promote the overall well-being of physically disabled adolescents. The emergence of these new applications of text analytics and natural language processing demonstrates the potential of these two fields to develop medical practises and foster inclusivity in society.

In another application of text analytics can be seen in the published works by Sang and Chen. This research group has leveraged the text analytics capabilities in developing an intelligent and fully automated physical education teaching mode. The human-computer interaction based on speech recognition technology was combined with physical education teaching. The research demonstrated that the voice cues from the students can be analysed and recognised by their proposed system which then sends information back to the students in different ways. This work implements the Mel cepstral coefficient technique for processing the external speech signal. The promising results from this research has enabled the emergence of human-computer interaction to cultivate active learning.

The extensive works conducted by Eom and Byeon shed light on the significant impact of COVID-19 pandemic on obesity trends in Korea. The research makes good use of text analytics and natural language processing techniques in order to extract useful insights from massive amounts of news data. The findings underscore the dynamic character of public health concerns and the significance of regularly monitoring and tackling developing factors that contribute to obesity. The findings also emphasise the fact that public health concerns are constantly evolving. The incorporation of such applications of text analytics and natural language processing into healthcare has the potential to facilitate the making of decisions based on evidence and assist in more effectively addressing ever-evolving health concerns.

During COVID-19 pandemic has also nurtured related research on text analytics and NLP. A scoping review of the existing landscape of AI-based applications in clinical trials was undertaken in one of the articles that was published in this Research Topic. The findings of this study highlight the growing applicability of text analytics and natural language processing in the medical field, particularly in light of the COVID-19 pandemic. The relevance of social media platforms as powerful sources of public opinion and emotional expression is highlighted in the study. The proposed approach uses techniques from sentiment analysis and machine learning to enable the recognition of real news relating to COVID-19 in Arabic language, with a particular emphasis on Gulf countries. The findings, which revealed that a pervasive negative feeling was experienced throughout the epidemic, underscore the need of monitoring public emotions and opinions in order to support effective policymaking and control efforts. These newly emerging uses of text analytics and natural language processing provide insightful information for healthcare professionals and policymakers, which makes it easier to implement timely interventions and response tactics during times of public health crises.

To summarise, the increasing applications of text analytics and NLP show a great deal of potential in changing the processes involved in providing medical care. By utilising these technologies, medical professionals and policymakers are able to improve their ability to make judgements, obtain more in-depth insights, and, as a result, improve the quality of treatment that is provided to patients. This has the potential to have a positive impact on the outcomes for patients as well as contribute to the general improvement of the field of healthcare in general.
